# Prevalence of Multidrug Resistance and Extended-Spectrum *β*-Lactamase Carriage of Clinical Uropathogenic *Escherichia coli* Isolates in Riyadh, Saudi Arabia

**DOI:** 10.1155/2018/3026851

**Published:** 2018-09-16

**Authors:** Abdulaziz Alqasim, Ahmad Abu Jaffal, Abdullah A. Alyousef

**Affiliations:** ^1^Microbiology Research Group, Department of Clinical Laboratory Sciences, College of Applied Medical Sciences, King Saud University, P.O. Box 10219, Riyadh 11433, Saudi Arabia; ^2^Department of Clinical Laboratory Sciences, King Saud Bin Abdulaziz University for Health Sciences, Riyadh, Saudi Arabia

## Abstract

The prevalence of extended-spectrum *β*-lactamase-producing *Escherichia coli* (ESBL-producing *E. coli*) has recently increased worldwide. This study aims at determining the antimicrobial susceptibility patterns of a collection of clinical *E. coli* urine isolates and evaluating the ESBL carriage of these isolates at phenotypic and genotypic levels. A total of 100 *E. coli* urine isolates were collected at a tertiary healthcare centre in Riyadh from January 2018 to March 2018. Antimicrobial susceptibility testing was carried out for all isolates. ESBL production was characterized at phenotypic and genotypic levels using double-disc synergy test and polymerase chain reaction, respectively. Detection of different ESBL variants was performed using DNA sequencing. Of 100 *E. coli* isolates, 67 were associated with multidrug resistance (MDR) phenotype. All isolates showed variable resistance levels to all antibiotics used here expect to imipenem, where they were all imipenem-sensitive. 33 out of 100 *E. coli* isolates were positive for ESBLs by phenotypic and genotypic methods. Among all ESBL-positive *E. coli* isolates, the CTX-M was the most prevalent ESBL type (31/33 isolates; 93.94%). CTX-M-15 variant was detected in all isolates associated with CTX-M carriage. Multiple ESBL gene carriage was detected in 15/33 isolates (45.45%), where 11 (33.33%) isolates produced two different ESBL types while 4 isolates (12.12%) associated with carrying three different ESBL types. Our study documented the high antimicrobial resistance of ESBL-producing *E. coli* to many front-line antibiotics currently used to treat UTI patients, and this implies the need to continuously revise the local guidelines used for optimal empirical therapy for UTI patients. It also showed the high prevalence of ESBL carriage in *E. coli* urine isolates, with CTX-M-15 being the most predominant CTX-M variant.

## 1. Introduction

Urinary tract infections (UTIs) are among the most common bacterial infections acquired in community and hospital settings [1]. UTIs are a main cause of hospital admissions and are associated with high morbidity, mortality, and economic costs [2, 3]. Worldwide, it is estimated that UTIs affect about 150 million people each year, costing the global economy more than 6 billion US dollars [4]. UTIs occur in all age groups and in both genders [5]. However their incidence increases with age [6], and the annual incidence of UTIs in the elderly population ranges from 10% in the community to as high as 30% of hospitalized patients [2]. UTIs are more common in women than in men [7], with an estimated 50–60% of women suffering from at least one UTI during their lifetime [3].

It is well known that *Escherichia coli* (*E. coli*) is the main causative agent of UTIs [8], and the uropathogenic *E. coli* (UPEC) group have the capability of causing around 90% of community-acquired UTIs and up to 50% of nosocomial UTIs [9, 10]. Over the past two decades, UPEC strains have shown an increased level of antimicrobial resistance to front-line antibiotics such as trimethoprim-sulfamethoxazole and ciprofloxacin. Several surveillance studies during the 2000s across Europe, North America, and South America have demonstrated that resistance to these antibiotics has been observed in as many as 20–45% of UPEC isolates [1, 11].

UPEC have also been associated with a high level of extended-spectrum *β*-lactamase (ESBL) gene carriage [12]. ESBLs comprise many plasmid-mediated derivatives such as TEM, OXA, and SHV [13]. Since 2000, a new group of ESBLs, called CTX-M (i.e., “active on CefoTaXime, first isolated in Munich”), has emerged [14]. Since then, CTX-M *β*-lactamases have been the predominant ESBL type worldwide [15]. Within the CTX-M family, CTX-M-15 is currently the most prevalent CTX-M genotype [14, 15]. This group of ESBLs has been associated with an extensive pattern of antimicrobial resistance to many antibiotics, including *β*-lactam agents such as penicillins, cephalosporins, monobactams, and carbapenems [16–19]. In addition, CTX-M-producing *E. coli* strains are often associated with coresistance to other large antimicrobial families such as aminoglycosides and fluoroquinolones [19]. This increasing level of antimicrobial resistance of UPEC is of great concern since it can limit the therapeutic choices used for treating common bacterial infections such as UTIs and highlights the growing threat of the emergence of pandrug resistance in UPEC.

Since treatment of UTI is frequently started empirically, prior information on the prevalence of causative agents as well as the antimicrobial susceptibility profiles in a particular setting is essential [20]. However, the distribution of UTI causative agents and their antibiotic susceptibility profiles differs regionally [21, 22]. Therefore, the evaluation of the local etiology and antimicrobial susceptibility profiles is essential to achieve the most effective empirical therapy [8].

In Saudi Arabia, previous studies have been conducted to determine the prevalence of ESBL-producing *Enterobacteriaceae* (EPE), with CTX-M enzymes being the most common ESBL type [23–30]. However, there are a limited number of studies on the prevalence of ESBL-producing *E. coli*. Additionally, there are few detailed reports on characterizing the genotypes of ESBL enzymes have been published.

This study aims at determining the antimicrobial susceptibility patterns of a collection of clinical *E. coli* urine isolates, to phenotypically assess the ESBL carriage of these isolates and to characterize the ESBL genotypes of these isolates. This study is important for providing clinicians with information required to facilitate the effective treatment and management of UTI patients.

## 2. Materials and Methods

### 2.1. Bacterial Isolates

One hundred nonconsecutive, nonduplicate clinical *E. coli* urine isolates were obtained from urine samples of inpatients hospitalized at a tertiary healthcare centre in Riyadh, Saudi Arabia. Midstream clean catch urine samples showing significant bacterial growth, of >10^5^ colony-forming units (CFU/mL) with a single type of bacteria, were considered positive for UTIs. Urine isolates were collected over a period of 3 months from January 2018 to March 2018 and were identified as *E. coli* using the Vitek 2 identification system (Vitek2-ID-GNB, BioMerieux). The isolates were stored at −80°C in Luria Bertani broth (HiMedia Labs, India) with 20% glycerol (v/v).

### 2.2. Antimicrobial Susceptibility Testing

Antimicrobial susceptibility testing (AST) for all isolates was carried out on Mueller–Hinton agar (HiMedia Labs, India) using disk diffusion method according to the guidelines of Clinical and Laboratory Standards Institute (CLSI) [31] using a panel of 10 commercially available antibiotics (LIOFILCHEM, Italy). Information on these antibiotics and their concentrations are shown in Table 1. *E. coli* ATCC 25922 was used as a control strain.

### 2.3. Phenotypic Detection of ESBL Production

ESBL production was assessed using the CLSI recommendations for ESBL screening and phenotypic confirmation tests [31]. For initial ESBL screening, UPEC isolates showing an inhibition zone size of ≤22 mm with ceftazidime (30 *μ*g) were identified as potential ESBL producers. The double-disc synergy test (DDST) was carried out for the phenotypic confirmation of ESBL production. For this test, a ceftazidime disc (30 *μ*g) was placed 25 mm away from a combination disc containing ceftazidime-clavulanic acid (30/10 *μ*g). When the zone of inhibition between the combination disc and the corresponding single antibiotic disc differed by ≥5 mm, the strain was identified as an ESBL producer. *E. coli* ATCC 25922 and *Klebsiella pneumoniae* ATCC 700603 were used as negative and positive control strains, respectively.

### 2.4. Polymerase Chain Reaction (PCR) of ESBL-Encoding Genes

Bacterial genomic DNA was extracted using the ONE-4-ALL Genomic DNA Mini-Preps Kit (Bio Basic Inc., Canada) according to manufacturer's instructions. PCR assays were performed to determine the presence of ESBL-encoding genes: *bla*_*OXA*_, *bla*_*TEM*_, *bla*_*SHV*_, and *bla*_*CTX-M*_ Groups 1, 2 and 9 using multiplex PCR primer sets (Table 2) and conditions previously described [32].

### 2.5. DNA Sequencing for Identification of CTX-M ESBL Gene Variants

Automatic DNA sequencing for both strands of all PCR products was carried out using the 3730*xl* DNA analyzer (ThermoFisher Scientific, United States) to identify the CTX-M ESBL variants. These variants were identified by comparison with the sequences in GenBank (http://blast.ncbi.nlm.nih.gov/Blast.cgi).

## 3. Results

### 3.1. Demographic Characteristics of Study Population

A total of 100 urine samples were collected from inpatients during this study period. Among these samples, 76 (76%) belonged to female patients while 24 (24%) belonged to male patients. With regard to the age categories of patients, a total of 60 (60%) samples were collected from adults, 21 (21%) samples belonged to elderly population, and 19 (19%) were obtained from children. Demographic characteristics of patients are shown in Table 3.

### 3.2. Antimicrobial Susceptibility Profiles and ESBL Prevalence

Antimicrobial susceptibility testing results are shown in Table 4. Among all antibiotics tested in this study, imipenem was the most active agent as all *E. coli* isolates were imipenem-susceptible. Out of the 100 *E. coli* isolates, 92% were resistant to ampicillin, 55% to amoxicillin-clavulanic acid, and 12% to gentamicin. The resistance rates for ceftazidime, cefoxitin, tetracycline, and trimethoprim-sulfamethoxazole were 29%, 13%, 49%, and 54%, respectively. Additionally, 40% and 15% of the isolates were nonsusceptible to ciprofloxacin and nitrofurantoin, respectively.

With regard to ESBL production, initial ESBL screening demonstrated that 41% of all *E. coli* isolates had an inhibition zone size of ≤22 mm with ceftazidime (30 *μ*g) and therefore initially identified as potential ESBL producers. However, phenotypic confirmation of ESBL production by DDST showed that 33 (33%) of all isolates were confirmed to be ESBL producers, while 67 (67%) isolates were non-ESBL producers.

The susceptibility profiles for the ESBL-producing *E. coli* isolates are shown in Table 3. Of all the 33 *E. coli* isolates identified as ESBL producers, 29 (88%) were resistant to amoxicillin-clavulanic acid, 28 (85%) to ceftazidime, 27 (82%) to trimethoprim-sulfamethoxazole, and 25 (76%) to ciprofloxacin. Additionally, 9 (27%) isolates were resistant to gentamicin, 6 (18%) to cefoxitin, 23 (70%) to tetracycline, and 7 (21%) to nitrofurantoin. All tested isolates were susceptible to imipenem, while they were all ampicillin-resistant.

For non-ESBL-producing *E. coli* isolates, the antimicrobial resistance levels were lower than that of ESBL-producing isolates for all antibiotics tested expect imipenem. Of all the 67 non-ESBL-producing *E. coli* isolates, 59 (88%) isolates were resistant to ampicillin, 27 (40%) to trimethoprim-sulfamethoxazole, 26 (39%) to amoxicillin-clavulanic acid, and 26 (39%) for tetracycline. The antimicrobial resistance rates for other antibiotics ranged from 1.5% for ceftazidime to 22% to ciprofloxacin. All non-ESBL-producing *E. coli* isolates were imipenem-susceptible.

### 3.3. Multidrug Resistance (MDR) Phenotype of *E. coli* Isolates

**Figure 1:**
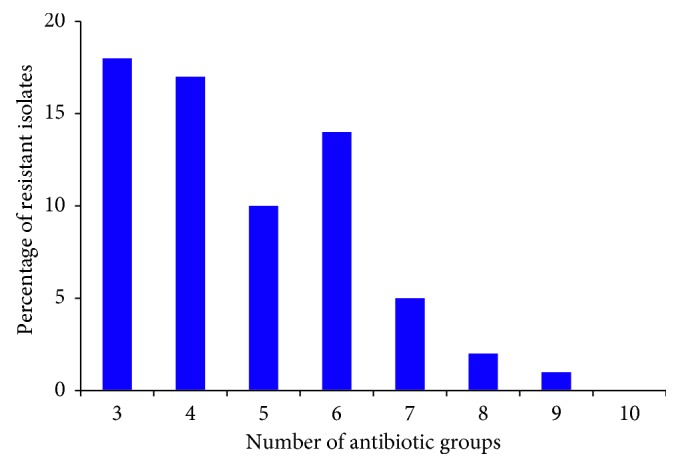
Percentage of clinical *E. coli* urine isolates showing multidrug resistance (MDR) phenotype.

Percentage of the clinical *E. coli* isolates showing MDR phenotype, which is defined as exhibiting resistance to at least 1 agent in ≥3 antimicrobial categories/groups [33], is shown in Figure 1. Our data showed a high level of multidrug resistance among the tested isolates with 67 (67%) of all *E. coli* isolates showing the MDR phenotype. Of these isolates, 18 (26.86%) isolates were resistant to 3 out of 10 antibiotic groups employed, 17 (25.37%) were nonsusceptible to 4 antibiotic groups, and 10 (14.92%) were resistant to 5 antibiotic groups. Additionally, 14 (20.89%), 5 (7.46%), and 2 (2.99%) were resistant to 6, 7, and 8 antibiotics, respectively. There was only 1 (1.49%) isolate that showed resistance to 9 antibiotics while none of the isolates had the ability to show resistance to all the 10 antibiotics used in this study. With respect to the association between ESBL production and the MDR phenotype, our data showed that all the 33 ESBL-positive *E. coli* isolates (100%) were MDR; however, only 34 out of the 67 non-ESBL-producing isolates (50.7%) were MDR.

### 3.4. Distribution of ESBL Gene Carriage in E. coli Isolates

33 ESBL-producing *E. coli* isolates were tested for the production of the major ESBL genes (*bla*_*OXA*_ *, bla*_*TEM*_ *, bla*_*SHV*_, and *bla*_*CTX-M*_ Groups 1, 2, and 9) by PCR. These isolates were all tested positive for ESBL production. The ESBL types detected in the *E. coli* isolates belonged to the CTX-M-Group 1 family, TEM and OXA ESBL genes, while the SHV-type, CTX-M-Group 2, and CTX-M-Group 9 were not detected in any of the tested isolates. The detected ESBL types were either solely or concomitantly harbored by the 33 ESBL-producing *E. coli* isolates. Our data demonstrated that 31 (93.94%) isolates were found to carry the gene encoding for CTX-M-Group 1 and 4 isolates (12.12%) produced the TEM-type ESBL. The CTX-M-Group 1 ESBL was solely carried by 17 isolates, whereas a sole TEM carriage was detected in only 1 isolate.

However, the gene encoding for OXA-type ESBL was not solely detected in any of the tested isolates but rather it was concomitantly produced by 8 isolates (24.24%) that either carry the genes encoding for CTX-M-Group 1, TEM, or both. Additionally, among all ESBL-positive isolates, 15 (45.45%) isolates were associated with multiple ESBL gene carriage. Of these, 11 (33.33%) were able to produce two different ESBL types, while 4 (12.12%) were able to concomitantly produce all the three ESBL types detected in this study. With respect to the CTX-M-Group 1 gene variants, our data showed that the CTX-M-15 variant was present in all the CTX-M-Group 1 producing isolates (Table 5).

## 4. Discussion

The current emergence of multidrug-resistant *E. coli* is becoming a global concern, and infections caused by MDR ESBL-producing *E. coli* represent a major challenge to clinicians and public health worldwide [34]. This study focuses on determining the antimicrobial susceptibility patterns of 100 *E. coli* urine isolates and on characterizing the ESBL carriage of these isolates at both phenotypic and genotypic levels.

Among the 100 urine samples collected in this study, 76 (76%) belonged to female population while 24 (24%) belonged to male patients. The gender distribution of patients in our study is consistent with many previous local [35, 36] and international [37, 38] reports showing the high incidence of UTI in women compared to men across all age groups. It has been demonstrated that the high UTI incidence in women can be attributed to many reasons such as anatomical factors that allow quick access of bacteria to the bladder, poor hygiene, sexual activity, and use of contraceptives [39]. With respect to age group of patients, our data showed that 60% of all study population was adults, followed by elderly (21%). This is in agreement with a recent report demonstrating that UTIs were more common in adults than any other age category [36]. Additionally, adult women were among the most dominant patient group in our study, and they accounted for 46% of total patient number. This concurs with previous studies showing that adult women are 30 times more likely to develop a UTI than men, with almost half of them experiencing at least one UTI episode during their lifetime [3].

The antimicrobial susceptibility patterns were determined for all the 100 *E. coli* urine isolates. The ESBL-producing *E. coli* isolates showed higher levels of resistance to all antibiotics compared to the non-ESBL-producing isolates expect for imipenem, where all *E. coli* isolates tested in this study were imipenem-sensitive. This is not surprising given that ESBL-producing *E. coli* strains are frequently associated with coresistance to other antimicrobial agents such as aminoglycosides and fluoroquinolones [19]. The ESBL-producing *E. coli* showed the greatest resistance to ampicillin, amoxicillin-clavulanic acid, ceftazidime, trimethoprim-sulphamethoxazole, and ciprofloxacin. This finding is in agreement with many previous reports [30, 40–42] showing that ESBL-producing *E. coli* isolates were more resistant to trimethoprim-sulphamethoxazole, ciprofloxacin, and third-generation cephalosporins compared to non-ESBL-producing *E. coli*, although this resistance did not affect imipenem. This increased resistance levels of ESBL-producing *E. coli* isolates to some front-line antibiotics such as cephalosporins, trimethoprim-sulphamethoxazole, and ciprofloxacin, could be ascribed to the inappropriate and excessive use of these agents in the empirical treatment of UTIs [43]. It has been shown that these antibiotics are commonly used for the treatment of UTIs worldwide [44], and the currently reported increase in resistance trends among uropathogens, primarily *E. coli*, has not only led to high rates of morbidity and mortality [45], but it has also complicated the management of UTIs [46].

In an attempt to achieve the effective treatment of UTIs, several guidelines were reviewed, in the late 2000s, to reposition nitrofurantoin as first-line therapy for community-acquired and nosocomial uncomplicated lower UTI [47, 48]. With respect to *E. coli* resistance to nitrofurantoin, although previous reports showed that it was low in China (1.6%) [49] and in North America (1.1%) [50], it has been reported to be high in Latin American hospitals (13%) [51].

An important worrisome finding in this study is the high rate of resistance to nitrofurantoin (21%) in the ESBL-producing *E. coli*. This is in contrary to many previous local [28] and international studies [49, 50] showing a low prevalence of nitrofurantoin resistance in *E. coli* isolates. The possible explanation of this high nitrofurantoin resistance is the currently reported exponential global increase in nitrofurantoin prescribing for the empirical treatment of hospitalized patients with positive urine cultures [52].

Based on the suggestion that if the resistance to a particular antibiotic is higher than 20%, that antibiotic should not be prescribed in the empirical antimicrobial treatment [53], and since our data show that the resistance levels of ESBL-producing *E. coli* isolates to 9 out of 10 antibiotics were more than 20%, we propose that carbapenems, such as imipenem, could be the drug of choice to treat UTI patients given that all *E. coli* isolates tested in this study were fully sensitive to imipenem, and this is the case with many recently published reports in Saudi Arabia showing *E. coli* urine isolates with full sensitivity to carbapenems [30, 54].

Additionally, we believe that it is crucial to assess the local antimicrobial resistance rates for specific pathogens and to revise the current guidelines used for optimal treatment regimens for ESBL-producing uropathogens, particularly *E. coli*, causing uncomplicated UTI in order to achieve the effective treatment.

Considering the level of multidrug resistance of *E. coli* isolates, this study found that 67 (67%) of all *E. coli* isolates were MDR. This is considered a high level of multidrug resistance when compared to a recently published report from Saudi Arabia showing that the MDR phenotype was determined in only 22.8% of all tested *E. coli* isolates [36]. Additionally, multidrug resistance was higher in ESBL-producing *E. coli* compared to non-ESBL *E. coli*, which is in total agreement with a previous local study showing the same finding [28], although the sample size here was low compared to that study.

Drug resistance of *E. coli* is mediated by ESBLs, mainly of the CTX-M family [19]. According to the Pan European Antimicrobial Resistance using Local Surveillance (PEARLS) study (2001–2002), the percentages of ESBL production among *E. coli* was 5.4% for all the study geographical sites [55]. Worldwide, many previous studies have shown that the prevalence of ESBL-producing *E. coli* varies across different geographical regions with low rates of about 1.5% reported in Denmark [56] and 5% in Canada [57] compared to much higher prevalence rates documented in reports from Turkey (36.7%) [58] and India (69%) [59].

In our study, we found that 33 (33%) isolates were ESBL producers, while 67 (67%) isolates were non-ESBL producers. The ESBL types detected in the *E. coli* isolates belonged to the CTX-M-Group 1 family, TEM and OXA, while the SHV-type, CTX-M-Group 2 and CTX-M-Group 9 were not detected in any of the tested isolates. Of these 33 ESBL-producing isolates, 31 (93.94%) were found to carry the gene encoding for CTX-M-Group 1, and DNA sequencing data showed that the CTX-M-15, a variant of CTX-M-Group 1, was detected in all of these isolates. Additionally, among all ESBL-positive isolates, 15 (45.45%) isolates were associated with multiple ESBL gene carriage. Of these, 11 (33.33%) were associated with a concomitant production of two different ESBL types, while 4 (12.12%) were associated with a concomitant production of all the three ESBL types detected in this study.

In this regard, previous studies in Saudi Arabia showed that the prevalence rates of ESBL-producing *E. coli* were 6.5% and 10.3% in 2002 and 2004, respectively [25]. However, recent reports have demonstrated a higher prevalence of ESBL-producing *E. coli* causing UTI. A 2-year retrospective observational study on the role of ESBL-producing *E. coli* in UTIs found that 27.4% of all *E. coli* strains isolated from urine samples were ESBL producers [60]. Another study found that the prevalence of ESBL-producing *E. coli* was 33.3% [28]. However, these studies did not determine the ESBL types present in *E. coli* isolates. Al-Agamy and colleagues determined the ESBL carriage and types of a collection of *E. coli* isolates and found that 20.4% of the total *E. coli* isolates were ESBL producers. They also found that CTX-M-15 was detected, concomitantly with the narrow-spectrum *β*-lactamase TEM-1, in all ESBL-producing *E. coli* [30].

Currently, CTX-M enzymes have replaced the traditional ESBL types such as SHV and TEM enzymes as the most prevalent ESBL type in *E. coli* [15]. Our study clearly shows that CTX-M enzymes are the dominant ESBLs in Saudi Arabia and that the CTX-M-15 was the most common variant among all CTX-M-Group 1, which is consistent with the previous finding reported by Al-Agamy and colleagues [30].

It has been shown that the successful dissemination of CTX-M-15 enzyme has been attributed to the spread of genetic elements, through horizontal gene transfer, and the clonal expansion of a pandemic *E. coli* clone, *E. coli* ST131 [19]. *E. coli* ST131 has been reported worldwide as the most common extraintestinal pathogenic *E. coli* (ExPEC) clone, and this clone is often MDR and is commonly associated with carrying CTX-M-15 enzyme [61, 62]. In the future, it would be important to study the molecular epidemiology of MDR *E. coli* strains to determine the population structure and clonal diversity of currently emerging MDR *E. coli* clones.

Our study has limitations: firstly, it used a low number of *E. coli* isolates that were obtained from a single healthcare facility in Saudi Arabia. Additionally, this study was performed on urine samples collected from hospitalized patients, which affects the accurate assessment of epidemiological changes in the community. Also important is that our study was performed in Riyadh City and this does not necessarily reflect the antimicrobial resistance trends in other regions within Saudi Arabia and that the clinical information of patients included in this study are very scarce.

However, this study shows a high prevalence of ESBL-producing *E. coli* compared to what has been previously reported in Saudi Arabia, with CTX-M-type enzymes being the most predominant ESBL type. Furthermore, our results provide evidence for the predominance of CTX-M-15-producing *E. coli* among UPEC isolates in Saudi Arabia, and this agrees with many reports demonstrating the changing epidemiology of ESBL-producing *E. coli* worldwide.

To conclude, this study demonstrates that *E. coli* isolates are associated with high multidrug resistance rates as well as high ESBL carriage. It also shows that ESBL-producing *E. coli* have high resistance levels to antibiotics, particularly to those used for empirical therapy of UTI patients. This highlights the need to evaluate the local antimicrobial resistance rates for specific pathogens and to review the current guidelines used for empirical treatment regimens for ESBL-producing uropathogens in order to ensure effective treatment of infections caused by these pathogens.

## Figures and Tables

**Table 1:** Details on antibiotics used in this study.

Antibiotic group	Antibiotic name	Concentration (*μ*g/disc)
Penicillins	Ampicillin (AM)	10 *μ*g
*β*-lactam/*β*-lactamase inhibitors combination	Amoxicillin-clavulanic acid (AUG)	20/10 *μ*g
Aminoglycosides	Gentamicin (GM)	10 *μ*g
Second-generation cephalosporins	Cefoxitin (FOX)	30 *μ*g
Third-generation cephalosporins	Ceftazidime (CAZ)	30 *μ*g
Tetracyclines	Tetracycline (T)	30 *μ*g
Folate pathway inhibitors	Trimethoprim-sulfamethoxazole (SXT)	1.25/23.75 *μ*g
Carbapenems	Imipenem (IMP)	10 *μ*g
Fluoroquinolones	Ciprofloxacin (CIP)	5 *μ*g
Nitrofurans	Nitrofurantoin (NI)	300 *μ*g

**Table 2:** Multiplex PCR-specific primers used for ESBL gene detection in *Enterobacteriacaea*.

PCR name	*β*-lactamase targeted	Sequence (5′– 3′)	Product (bp)	Reference
Multiplex I	TEM variants including TEM-1 and TEM-2	CATTTCCGTGTCGCCCTTATTC	800	[32]
		CGTTCATCCATAGTTGCCTGAC		
	SHV variants including SHV-1	AGCCGCTTGAGCAAATTAAAC	713	
		ATCCCGCAGATAAATCACCAC		
	OXA-1, OXA-4, and OXA-30	GGCACCAGATTCAACTTTCAAG	564	
		GACCCCAAGTTTCCTGTAAGTG		
Multiplex II	Variants of CTX-M group 1 including CTX-M-1, CTX-M-3, and CTX-M-15	TTAGGAARTGTGCCGCTGYA	688	
		CGATATCGTTGGTGGTRCCAT		
	Variants of CTX-M group 2 including CTX-M-2	CGTTAACGGCACGATGAC	404	
		CGATATCGTTGGTGGTRCCAT		
	Variants of CTX-M group 9 including CTX-M-9 and CTX-M-14	TCAAGCCTGCCGATCTGGT	561	
		TGATTCTCGCCGCTGAAG		

**Table 3:** Demographic data of study population.

Age category	Gender		Total
	Female	Male	
Children^*∗*^	16	3	19
Adults^*∗∗*^	46	14	60
Elderly^*∗∗∗*^	14	7	21
Total	76	24	100

^*∗*^Children: 0–17 years, ^*∗∗*^adults: 18–64 years, ^*∗∗∗*^elderly: ≥65 years.

**Table 4:** Antibiotic resistance rates of *E. coli* isolates tested in this study.

Antibiotic	Number (%) of resistant isolates		
	ESBL producing (*n*=33)	Non-ESBL producing (*n*=67)	Total (*n*=100)
Ampicillin	33 (100%)	59 (88%)	92 (92%)
Amoxicillin-clavulanic acid	29 (88%)	26 (39%)	55 (55%)
Gentamicin	9 (27%)	3 (4.5%)	12 (12%)
Ceftazidime	28 (85%)	1 (1.5%)	29 (29%)
Cefoxitin	6 (18%)	7 (10.5%)	13 (13%)
Tetracycline	23 (70%)	26 (39%)	49 (49%)
Trimethoprim-sulfamethoxazole	27 (82%)	27 (40%)	54 (54%)
Imipenem	0 (0%)	0 (0%)	0 (0%)
Ciprofloxacin	25 (76%)	15 (22%)	40 (40%)
Nitrofurantoin	7 (21%)	8 (12%)	15 (15%)

**Table 5:** The distribution of *β*-lactamase genes among ESBL-producing *E. coli* isolates.

Isolate ID	*β*-lactamase types						CTX-M G1 variants
	CTX-M G1	CTX-M G2	CTX-M G9	TEM	OXA	SHV	
U1	+	−	−	−	−	−	CTX-M-15
U4	+	−	−	−	−	−	CTX-M-15
U7	+	−	−	−	−	−	CTX-M-15
U9	+	−	−	−	−	−	CTX-M-15
U10	+	−	−	−	−	−	CTX-M-15
U11	+	−	−	−	−	−	CTX-M-15
U12	+	−	−	−	−	−	CTX-M-15
U15	+	−	−	−	+	−	CTX-M-15
U16	+	−	−	−	−	−	CTX-M-15
U17	+	−	−	−	−	−	CTX-M-15
U20	+	−	−	−	−	−	CTX-M-15
U24	+	−	−	−	−	−	CTX-M-15
U27	+	−	−	−	−	−	CTX-M-15
U28	+	−	−	−	+	−	CTX-M-15
U31	+	−	−	−	+	−	CTX-M-15
U46	+	−	−	−	+	−	CTX-M-15
U55	+	−	−	+	+	−	CTX-M-15
U57	+	−	−	−	−	−	CTX-M-15
U63	+	−	−	−	+	−	CTX-M-15
U65	+	−	−	+	+	−	CTX-M-15
U66	+	−	−	+	−	−	CTX-M-15
U68	+	−	−	−	−	−	CTX-M-15
U71	+	−	−	−	−	−	CTX-M-15
U73	+	−	−	−	+	−	CTX-M-15
U74	+	−	−	−	−	−	CTX-M-15
U75	−	−	−	+	−	−	NA^1^
U78	+	−	−	−	−	−	CTX-M-15
U82	+	−	−	+	+	−	CTX-M-15
U85	+	−	−	+	+	−	CTX-M-15
U87	+	−	−	−	+	−	CTX-M-15
U93	+	−	−	+	−	−	CTX-M-15
U95	−	−	−	+	+	−	NA
U98	+	−	−	+	−	−	CTX-M-15

^1^Nonapplicable.

## Data Availability

All data generated or analyzed during this study are included in this published article and its supplementary information files or available from the corresponding author upon request.

## Abbreviations

ESBL-producing *E. coli*: Extended-spectrum *β*-lactamase-producing *Escherichia coli*
MDR: Multidrug resistance
UTIs: Urinary tract infections
UPEC: Uropathogenic *E. coli*
CTX-M: “Active on CefoTaXime, first isolated in Munich”
TEM: ESBL variant isolated from a patient named Temoneira
SHV: Sulfhydryl variable
OXA: Oxacillin hydrolyzing capabilities
CLSI: Clinical and Laboratory Standards Institute
DDST: Double-disc synergy test
AST: Antimicrobial susceptibility testing
PCR: Polymerase chain reaction
*μ*g: Microgram
ST: Sequence type.

## Supplementary Materials

Supplementary Materials All data generated or analyzed during this study are included in this published article and in the supplementary materials attached. Supplementary materials include the results of antimicrobial susceptibility testing of *E. coli* isolates.
